# Small-scale Farmer Pesticide Knowledge and Practice and Impacts on the Environment and Human Health in Ethiopia

**DOI:** 10.5696/2156-9614-11.30.210607

**Published:** 2021-05-28

**Authors:** Mekuria Teshome Mergia, Ermias Deribe Weldemariam, Ole Martin Eklo, Girma Tilahun Yimer

**Affiliations:** 1 Hawassa University, Department of Biology, Hawassa, Ethiopia; 2 Kotebe Metropolitan University (KMU), Addis Ababa, Ethiopia; 3 Norwegian University of Life Science (NMBU), Department of Environmental Sciences and Natural Resource Management, Ås, Norway

**Keywords:** Lake Ziway, pesticide, small-scale farmers

## Abstract

**Background.:**

Inappropriate use and application of pesticides in Ethiopia pose a major threat to the health of farmers and the environment.

**Objective.:**

The present study aimed to assess the level of knowledge, attitudes, and practices of small-scale vegetable farmers towards the use of pesticides in Ethiopia along the Lake Ziway watershed.

**Methods.:**

This was a cross-sectional study involving a total of 210 farmers randomly selected during a period of pesticide application from a purposively selected village located in the immediate vicinity of Lake Ziway, Ethiopia. Data were generated through structured in-depth interviews and on-site observations on farms. A Chi-square test was applied to evaluate whether the collected data and their probable associations were significant.

**Results.:**

World Health Organization (WHO) class II pesticides (moderately toxic) were the most frequently used pesticides in the study area. There was no reported use of WHO classes 1a and 1b and banned or restricted pesticides such as dichlorodiphenyltrichloroethane (DDT) and endosulfan. Most (92%) farmers reported indiscriminately disposing of empty containers in the field, while 86.7% applied the leftover pesticides to other crops. More than 90% of small-scale farmers did not use any personal protective equipment (PPE) when handling pesticides. About 95% of farmers had poor knowledge regarding pesticides. A significant association (p < 0.001) was observed between the knowledge of farmers and their practices related to pesticides.

**Conclusions.:**

Generally, the knowledge of small-scale farmers on pesticides was poor. Moreover, the inappropriate disposal of pesticides and pesticide containers poses a risk to the environment. The findings of the present study underline the need to train farmers concerning the safe and proper use of pesticides to mitigate hazards to human health and the environment.

**Participant Consent.:**

Obtained

**Ethics Approval.:**

The study was granted an exemption from requiring ethics approval from the Hawassa University College of Natural and Computational Science, Research and Review Committee.

**Competing Interests.:**

The authors declare no competing financial interests.

## Introduction

**Figure 1:**
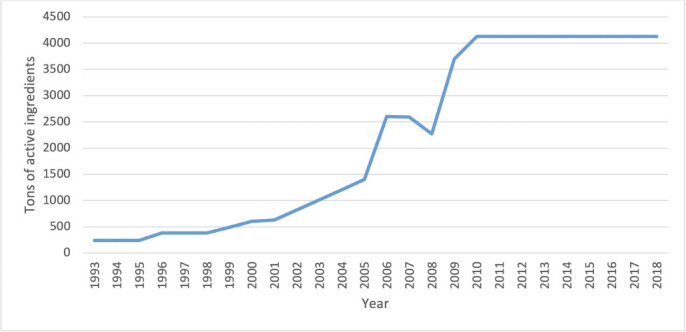
Pesticide use in Ethiopia (1993–2018) from FAOSTAT database^2^

Pesticides are essential chemicals that are used to protect against crop damage and improve agricultural output.^1^ The use of pesticides was introduced in the 1960s in Ethiopia to smallholder farmers through agricultural extension systems. The use of pesticides has shown steady growth and with the current development of the flower growing sector, average imports of pesticides have grown to over 2400 tons per annum.^2^ From 2005 to 2010, the import and use of pesticides in Ethiopia has grown rapidly *(Figure 1).* However, the negative effects of pesticide use in vegetable cultivation have been widely reported.^1^ A great majority of pesticides are used for pest and vector control in farming areas, but many farming societies around Lake Ziway, Ethiopia are not adequately informed of the hazards related to pesticide usage.^3^ Consequently, farmers use pesticides without knowledge of their impact on human health and the environment. Even though many vegetable farms along the Lake Ziway watershed depend on irrigation,^4^ their proximity to water bodies could make the use of pesticides problematic. Pesticide use in vegetable growth negatively affects both the surrounding environment and human health and farmer productivity.^3^ Numerous studies in Africa have shown misuse and improper handling of pesticides and poor knowledge of pesticides among farmers in general.^5^,^6^,^7^,^8^ This lack of awareness of proper pesticide management practices poses a risk to human health and the environment.

Ensuring environmental protection and sustainability of pesticide supply and use are fundamental challenges of pesticide administration.^9^ Likewise, misuse and poor management of pesticides in Ethiopia are increasing due to the rapid growth of large-scale farms.^10^,^11^ Currently, Ethiopia is the second-largest flower exporting country in Africa, and the sector is growing rapidly. Despite its economic advantages, the state of flower production has made the sector susceptible to criticism about working conditions and environmental impact.^10^,^11^,^12^ Because of the rapid growth of agricultural activities along the Lake Ziway watershed, it currently has a high anthropogenic burden.^2^,^13^ The expansion of agricultural activities around the lake has led to an increase in intensive and unplanned uses of pesticides and fertilizers and therefore added to the increasing degradation of the lake's water quality.^10^ The misuse of pesticides also has undesirable effects on human health, leading to headache, rashes, disorientation, shock, nausea, vomiting, respiratory failure, and even death. To the best of our knowledge, there are no formerly available studies on the level of knowledge and practice of pesticide use and associations among small-scale farmers near Lake Ziway in Ethiopia. The present study aimed to investigate pesticide utilization in small-scale vegetable farm fields along the Lake Ziway watershed and to determine how they impact the health of vegetable farmers and the environment. The present study focused on the lakeshore adjacent to where vegetables are grown, to determine whether pesticide usage in this area has any persistent biological effects.

**Abbreviations**
*IPM*: Integrated pest management
*PPE*: Personal protective equipment

## Methods

The study took place along the Lake Ziway watershed located in the Rift Valley region in Ethiopia, which is a major vegetable growing area. The districts holding the lakes shoreline are Adami-Tulu-Jido-Kombolcha, Dugda, and Ziway Dugda. Tomato, cabbage, and onion are the major crops in this area. Farming activities have considerably transformed the area operationally and economically. Water from Lake Ziway is used for the irrigation of these crops. In order to boost production, small-scale farmers often practice intensive usage of fertilizers and pesticides. Vegetables produced in this cultivation framework are for export purposes and local consumption in Addis Ababa (the capital city of Ethiopia).^10^ These intensive agricultural activities have put the lake in danger of agrochemical contamination. Furthermore, Lake Ziway is under threat of increasing urbanization, because the lake is situated adjacent to the fast-growing towns of Ziway (recently called Batu) to the southwest and Meki to the northwest.^14^

The survey study was conducted from October to March 2019 in 12 vegetable-growing irrigated villages (the smallest rural administrative unit) of the Adami-Tulu-Jido-Kombolcha (Ziway area) and Dugda (Meki area) districts in the Central Rift Valley, Ethiopia. These districts were selected because the majority of small-scale farmers in these areas cultivate vegetables with the use of pesticides and are situated along Lake Ziway. Village leaders and agricultural experts who cultivate vegetables that require the use of pesticides were consulted when the study group was selected. The present study employed a structured and semi-structured questionnaire that was established according to similar published studies.^32^

### Sampling procedures

A cross-sectional study involving a total of 210 randomly selected small-scale farmers was interviewed from purposively selected vegetable growing villages located along the Lake Ziway watershed. Respondents were household members who grew vegetable crops. There were a total of 930 households in the selected villages and the lists of households who used irrigation were obtained from their respective village (*kebele*) administration, District Offices of Irrigation Development, and Office of Cooperative Promotion. Respondents were allocated equally from each village and the determined sample size (210) was proportionally selected from all villages. Respondents were selected from each village randomly through simple random sampling without replacement using the lottery method. The sample size was determined according to the formula of Leslie^15^ and proportionally selected from each village. Primary sample gathering was done by fieldwork for all three studies. Interviews of farmers, key informant interviews, and field observations were performed. This mixed-method approach aids triangulation and increases the rationality and dependability of the results.^16^ Face-to-face interviews were piloted to collect samples from farmers. The questionnaire was prepared in English and translated into Amharic and local languages (Oromifa) by the researcher and field assistants (under the direction of the researcher). The sample size was obtained using Equation 1:

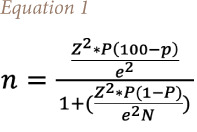
Where n = required sample size, z = 1.96 for 95% confidence level, p = percentage selecting a choice, e = the percentage maximum error required (margin of error), and N = population size (930).

The questionnaire (*Supplemental Material 1*) aimed to collect data in four categories. Part one collected general information on farmer demographics, vegetable growing experience, pesticide use over the past 5 years, and land tenure. The second part consisted of eight questions to evaluate the knowledge of small-scale farmers about pesticides. The answers were recorded as ‘yes', ‘no', or ‘don't know. For every ‘correct' answer, a mark of ‘+1' was given. For the ‘wrong' answer, a mark of ‘−1' and for ‘don't know, a mark of ‘0' was given. A score of four or more indicated good knowledge and fewer than four indicated poor knowledge.^17^ Next, pesticide use and practices were addressed to collect information on the kinds of pesticides used, pesticide sources, ability to read the information available on the pesticide label, pesticide use practices such as the method of mixing, rates, and amounts of pesticides sprayed, use of personal protective equipment (PPE), disposal of pesticide cans and application methods.^3^ Finally, social and ecological effects were addressed to gather data on the negative effects of pesticides exposure, for example, the symptoms frequently experienced before or after pesticide spraying procedures and altering trends in biodiversity, e.g. changes in pests, birds, and insects (whether increasing, decreasing or constant). To investigate public health problems, data were collected on intense side effects (symptoms) that showed up inside forty-eight hours of pesticide application.

Data regarding training and support to farmers were obtained by interviewing retailers and agriculture extension workers. Ten (10) retailers, five (5) government extension workers, and four (4) plant protection workers were interviewed to collect additional data as supportive qualitative information. These key informants were interviewed for information on training and support provided to farmers either by retailers or government extension workers. Pictures of important observations were also included as supportive qualitative data. Checklists were used as an aid during field observation. Photos were frequently taken to document how farmers store, mix and spray pesticides.

### Statistical analysis

The data were prearranged and entered in the MS-Excel spreadsheet and then analyzed using Statistical Package for the Social Sciences (SPSS) version 20. Chi-square test was applied to evaluate whether the collected data and their probable associations were significant or whether variables were related to each other. The results were presented in frequencies, and percentages for specific variables, and as mean ± SD for continuous variables. The significance levels were set at *P* ≤ 0.01 and *P* ≤ 0.05.

### Ethics approval

The study was granted an exemption from requiring ethical approval from the Hawassa University College of Natural and Computational Science, Research and Review Committee. The purpose and importance of the study were explained to the participants. Data were collected after full informed verbal consent was obtained and confidentiality of the information was maintained by using unique codes. Respondents were informed of their right to withdraw from the study at any time.

## Results

The demographic characteristics of small-scale farmers are shown in Table 1. More than 98% of small-scale farmers interviewed in the study were men, as farm activities and pesticide spraying are primarily performed by men in this area. Half of the small-scale vegetable farmers (50.5%) ranged between 31 and 54 years of age, with a mean of 35.2 years. Nearly 8% of the farmers were over 55 years old. A substantial number of the farmers (41.4%) had no education (illiterate), while 51.4% had an elementary school education (grades 1–8), and few (5.7%) farmers had completed secondary school, while the remaining (1%) had a college/university education. In addition, 45.7% of farmers had five to ten years of farming experience, and 41.9% had greater than ten years of farming experience. The respondents were typically small-scale farmers with farm sizes ranging from less than one hectare to over ten hectares with an average of 1 ha. Nearly 60% of farmers had land possessions smaller than 1.1 ha and 40% of the framers possessed land over 1.0 ha. The land used by 61.9% of the small-scale farmers was rented from local farmers for 3 to 5-year contracts. Also, 81.9% of farmers reported an increasing trend in pesticide use during the past five years, while 18% considered the situation to be constant and no one stated that pesticide use was decreasing. Only 4.2% of small-scale farmers received training on the proper use and safe handling of pesticides. However, 94% of farmers did not have any training.

**Table 1:** Demographic Characteristics of Small-Scale Farmers

**Variable**	**Respondents (N)**	**Percentage (%)**
Gender		
Male	206	98
Female	4	2
Age group		
18–30	86	41
31–54	106	50.5
>55	18	8.5
Education level		
None (Illiterate)	87	41.4
1 to 8 grades	108	51.4
9 to 12 grades	13	6
College/University	2	1
Farm sizes (ha)		
< 1 ha	126	60
> 1 ha	84	40
Land tenure situation		
landowners	80	38
landholders	130	61.9
Trend pesticide use past 5 years		
Increasing	172	81.9
Constant	38	18
Farming experience		
1–3 years	26	12.4
5–10 years	88	41.9
>10 years	96	45.7
Professional training		
Yes	9	4.3
No	201	95.7

### Types of pesticides used by small-scale vegetable farmers

Results indicate that a wide range of pesticides are used by small-scale farmers along the Lake Ziway watershed. A total of 28 pesticides (active ingredients) were reported in the study area. These pesticides are presented based on the WHO classification and chemical class in Supplemental Material 2, Table 1. The great majority of the pesticides (90%) used in the study area belonged to the WHO toxicity Class II (moderately hazardous), followed by Class-U (unlikely to present acute hazards in normal use and Class III (slightly hazardous). Extremely hazardous (Ia) or highly hazardous (Ib) classified pesticides were not reported by any of the farmers. The primary WHO Class II pesticides reported by small-scale farmers in the study area included metalaxyl (98%), profenofos (98%), dimethoate (95%), cypermethrin (93%), lambada-cyhalothrin (72%), and imidacloprid (52%). Among Class-U pesticides, mancozeb (100%) were the most reported pesticides. Herbicides like propanil and 2, 4-D were reportedly used but were not very common. The majority of the farmers along the Lake Ziway watershed used insecticides and fungicides on vegetables *(Supplemental Material 2, Table 1).* Insecticides and fungicides were utilized by 98% and 90% of farmers, respectively, while herbicides were used by only 3.8% of farmers. In general, limited herbicide use was reported by small-scale farmers.

### Knowledge and attitudes towards pesticides among small scale farmers

The small-scale farmers' level of knowledge of pesticides, routes of exposure, impact on the environment, health effects of pesticides, and awareness of pesticide policy and code of practice are summarized in Table 2. Nearly 69% of farmers did not read or follow directions on pesticide labels. Most farmers (95%) were not aware of the toxicity color codes present on the pesticide containers. In the present study, the most frequent routes of exposure to pesticides reported by farmers were dermal (17%), inhalation (50%), and oral (46%).

**Table 2:** Farmers' Knowledge, Attitude, and Understanding of Pesticides (n = 210)

**Variable**	**N**	**%**
Do you think that pesticides affect human health?		
yes	187	89
No	15	7.1
Don’t know	8	3.8
Do you think that pesticides affect the environment?		
yes	43	20
No	145	69
Don’t know	22	10
Do you think pesticides are indispensable for high crop yield?		
yes	205	98
No	1	0.5
Don’t know	4	1.9
Do you read, understand and follow pesticide labels		
yes	56	27
No	154	73
How do pesticides enter the human body? (Multiple answers possible)		
Dermal	36	17
Inhalation	106	50
Oral	96	46
Eye contact	97	46
Don’t know	36	17
Do you know the pesticides that are banned or restricted for use?		
Yes	16	7.6
No	194	92.2
Do you know the reasons for banning or restricting pesticides? (n=16)		
Highly toxic	9	56
Not effective	3	19
Expensive	2	13
Don’t know	2	13
Aware of the toxicity color codes present on the pesticide containers		
Yes	10	4.8
No	200	95

About 17% of farmers reported a lack of knowledge of any route of exposure to pesticides. The vast majority of small-scale farmers (92.4%) were not aware that some pesticides have been banned or restricted. Only 7.6% of the respondents knew the names of some of the pesticides that are prohibited or restricted. Of farmers who were aware that some pesticides were prohibited or restricted, 56% were aware of the potential high toxicity of pesticides: 19% of farmers described them as ineffective, 13% stated that they were prohibited or limited due to being expensive, and the remaining 13% stated that they did not know why some pesticides were prohibited or restricted. Overall, a total of 10 farmers had good knowledge, and the remaining 200 had poor knowledge. A significant association (X^2^ =77.82, p < 0.01) was observed between good knowledge and level of education *(Table 3).*

**Table 3:** Association Between Knowledge of Pesticides and Demographics of Farmers, and Pesticide Use Practice

**Variables**	**Good knowledge (n=10)**	**Poor knowledge (200)**	**Total (n=210)**	*******X^2^* value & p value**
Educational level				
None (Illiterate)	0	86 (43%)	86 (40.9%)	77.82
1–8	3 (30%)	106 (53%)	109 (51.9)	<0.001
9–12	5 (50%)	8 (4%)	13 (6.2%)	
University/College	2 (20%)	0	2 (1%)	
Age group (years)				2.01
18–30	3 (30%)	83 (41.5)	86 (40.9%)	0.368
31–54	7 (70%)	99 (49.5)	106 (50.5%)	
>55	0	18 (9%)	18 (8.6%)	
Farm experience (years)				0.79
1–3	2 (20%)	25 (12.5%)	27 (12.9%)	>0.05
5–10	5 (50%)	91 (45.5%)	96 (45.7%)	
>10	3 (30%)	84 (42%)	87 (41.4%)	
Source of pesticide information for purchase or usage				
Agrochemical retailers	28 (14%)	7 (70%)	35 (17%)	23.484
Other farmers	95 (47.5%)	2 (20%)	97 (46%)	<0.001
Previous experience	68 (34%)	0 (0%)	68 (34%)	
Agriculture extension officers	8 (4.5%)	1 (10%)	9 (4.3%)	
Source of pesticide dose information				
Agrochemical retailers	85 (42.5%)	1 (10%)	86 (41%)	26.21
Other farmers	64 (32%)	0	64 (30.5%)	<0.001
Previous experience/information on container	40 (20%)	9 (90%)	49 (23.3%)	
Agriculture extension officers	6 (3%)	0	6 (3%)	
Agriculture extension officers + retailers	5 (2.5%)	0	5 (2.5%)	
Place where pesticides were bought				
Agrochemical retailers	94 (47%)	9 (90%)	103 (49%)	7.89
General household shops	20 (10%)	1 (10%)	21 (10%)	0.019
Other farmers	86 (43%)	0	86 (41%)	

### Sources of pesticides and pesticide information

Table 3 presents the association between knowledge and farmers' sources of information concerning pest control and pesticide spray techniques. In the present study, 46% of small-scale farmers received relevant information regarding pest control and pesticide application practices via oral communication with other farmers, followed by previous experience (34%). About 4.3% of farmers stated that they had learned the information on pest control and pesticide application from government extension services. There was a significant association between the source of information on the proper use of pesticides and the knowledge of farmers (X^2^, 23.48, p < 0.001).

Regarding pesticide dosage, irrespective of category, small-scale farmers relied mostly on agrochemical retailers (41%), followed by other farmers (30.5%). The majority of the farmers with good knowledge (90%) reported that they received information by reading the instructions on the pesticide containers and previous experience. There was a significant association between the source of information on pesticide dosage and knowledge of small-scale farmers (X^2^ = 26.21, p < 0.001). Nearly half of respondents (49%) said they bought pesticides from agrochemical retailers, which is the principal source of pesticides in the study area, followed by other farmers (41%). Few farmers (10%) purchased pesticides from general household shops. The majority of farmers with good knowledge (90%) bought pesticides from agricultural chemical retailers. There was a significant association between where pesticides were bought and farmer knowledge (X^2^, 7.89, P =0.019) *(Table 3).* General household shops regularly repackaged products without labeling the container. According to the key informants (agricultural extension workers), informal traders repackaged pesticides into empty plastic water bottles made of polyethylene terephthalate or scraps of plastic, without labeling.

### Pesticide usage among small-scale farmers

All of the interviewed small-scale farmers (210, 100%) used pesticides on their farms. None of the vegetable farmers reported using biological methods or integrated pest management (IPM). They depended on the experience of other farmers to determine the activity level of pesticides. Nearly 86.6% of respondents reported not eating and 57.7% reported not drinking when blending or spraying pesticides *(Table 4).* Furthermore, 93.8% of farmers reported that they did not take a shower after mixing or spraying pesticides. Likewise, 64.7% of respondents stated that they did not take into account wind direction while spraying pesticides. A lever-operated knapsack sprayer was the only type of sprayer used by small-scale farmers. There was a significant association between farmers considering wind direction and knowledge of pesticide (X^2^=107.8, p < 0.001) *(Table 4).* Many small-scale farmers (40.5%) mixed directly in the sprayer (knapsack), but most farmers (59.5%) mixed pesticides in a separate blue drum container before transferring the mixture to a knapsack sprayer *(Table 5).* According to the key informants, the mixing containers are reused by the farmers for other activities, such as holding fruit and vegetables and storing drinking water.

**Table 4:** Association Between Farmers' Knowledge and Pesticide Use Practices

**Variable**	**Poor knowledge**	**Good knowledge**	**Total (n=210)**
Farmer uses pesticides			
Yes	200 (100%)	10 (100%)	210 (100%)
No	0	0	
Farmer uses biological pesticides or other forms of integrated pest management			
Yes	0	0	
No	200 (100%)	10 (100%)	210 (100%)
Farmer drinks while mixing or spraying			
Always	0	0.0	
Sometimes	9 (4.5%)	4 (40%)	13 (6.2%))
Never	191 (95%)	6 (60%)	197 (93.8%)
Farmers eat while mixing or spraying			
Always	0	0	
Sometimes	28 (14%)	0	28 (13.3%)
Never	172 (86%)	10 (100%)	182 (86.6%)
During the preparation of the different formulae, do you use standard pesticide quantification materials?			
Yes	71 (35.5%)	0	71 (35.5%)
No	129 (64%)	2 (20%)	131 (62.4%)
Sprayed with the wind direction			
Always	4 (2%)	8 (80%)	12 (5.7%)
Sometimes	36 (18%)	1 (10%)	37 (17.6%)
Never	160 (80%)	1 (10%)	161 (76.7%)
Frequency of pesticide application			
3–5 times per season	17 (8.5%)	8 (80%)	25 (11.9%)
7–10 times per season	64 (32%)	0	64 (30.5%)
12–15 times per season	95 (47.5%)	1 (10%)	96 (45.7%)
More than 15 times	24 (12%)	1 (10%)	25 (11.9%)

**Table 5:** Association Between Farmers' Knowledge and Pesticide Mixing Practices

**Variable**	**Poor knowledge**	**Good knowledge**	**Total (n=210)**
Showering immediately after mixing or spraying			
Always	0	0.0	
Sometimes	9 (4.5%)	4 (40%)	13 (6.2%))
Never	191 (95%)	6 (60%)	197 (93.8%)
Where are pesticides mixed?		0.0	
In the farm	116 (58%)	5 (5%)	121 (57.6%)
Near water /community water sources	63 (31.5%)	4 (40%)	67 (31.9%)
At home	21 (10.5%)	1 (10%)	22 (10.5%)
Devices used for mixing pesticides			
Knapsacks	84 (42%)	1 (10%)	85 (40.5%)
Blue containers (drum)	116 (58%)	9 (90%)	125 (59.5%)
How pesticides are mixed			
With a stick, but bare hands	182 (91%)	7 (70%)	189 (90%)
With bare hands	16 (8%)	0	16 (7.6%)
With hands and wearing gloves	2 (1%)	3 (30%)	5 (2.4%)

**Figure 2:**
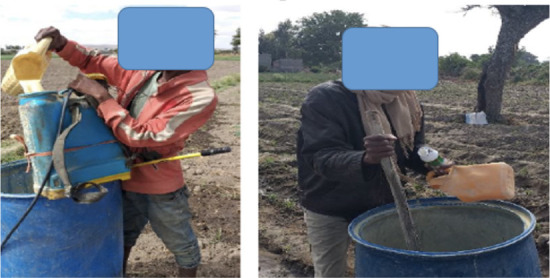
Small-scale farmers mixing pesticides with bare hands and with a stick and bare hands

More than half of the interviewed farmers mixed their pesticides on their farm (57.6%) and 31.9% of the farmers mixed near water a source (Lake Ziway). The remaining 10.5% formulated the pesticide mixture at home *(Table 5).* No significant association was observed between pesticide formulation trends and knowledge of farmers (P > 0.05). Farmers frequently used a stick and bare hands (90%) or bare hands only (7.6%) to mix pesticides *(Figure 2).* Only 2.4% of farmers wore gloves when mixing. Farmers with good knowledge of appropriate pesticide usage had a greater tendency to wear gloves while mixing pesticides compared to farmers with poor knowledge (X^2^ = 34.4, P < 0.001). While farmers did not keep records of the amount of pesticides sprayed, they mentioned that their application rate was widely variable, depending on weather conditions and crop types. A total of 45.7% of vegetable farmers mixed pesticides and sprayed 12 to 15 times per season, 30.5% sprayed 5 to 10 times per season, 11.9% sprayed 3 to 5 times per season, and 12.4% of farmers reported that they sprayed more than 15 times per season *(Table 5).* About 12.4% of farmers sprayed fungicides up to 20 times and insecticides up to 16 times per harvesting season. There was a significant association between spraying frequency and pesticide knowledge (X^2^=47.06, p < 0.001).

### Farmers' reports of pesticide poisoning symptoms

**Figure 3:**
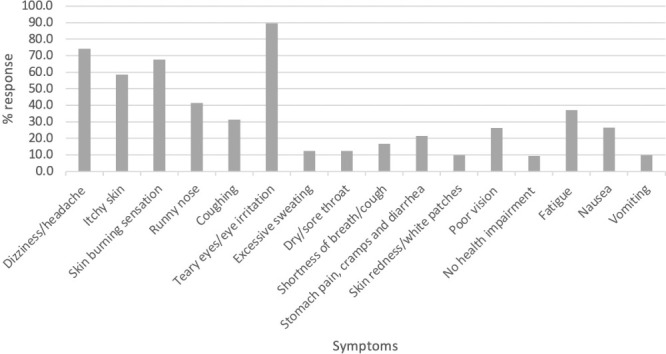
Effects of pesticide exposure reported by farmers during and after pesticide application (multiple answers possible)

The most commonly stated symptoms were headaches (74.3%), skin burning sensation (67.6%), itchy skin (58.6%), eyes irritation (89.5%), runny nose (41%), fatigue (37%), and coughing (31.4%). Farmers also reported symptoms such as fatigue (37.1%), nausea (26.7%), poor vision (26.2%), stomachache (21.4%), excessive sweating (12.4%), skin redness (10%), vomiting (10%), and sore throat (12.4%). Regarding the action they took following cases of poisoning, 75% of farmers stated did not take any action as the case was insignificant or required only self-treatment *(Figure 3).*

### Personal protective equipment

**Figure 4:**
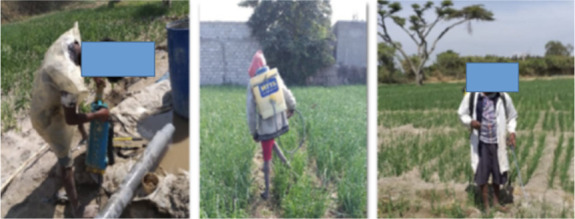
Small-scale farmers without improper PPE (bare feet, no facemasks, no gloves, wearing torn long-sleeved shirts and trousers) while spraying pesticides

Almost none of the small-scale farmers in the study area reported using PPE when mixing or spraying pesticides. There was a significant association between the use of PPE and the knowledge of farmers (P < 0.001) *(Table 6).* Around 4% of farmers reported not using PPE due to lack of accessibility, and 64.3% of respondents stated that using PPE is uncomfortable because of the hot and humid climate. About 39.5% of farmers reported that PPE is expensive and the other 43.8% reported that it slowed their work. The majority of farmers reported not wearing a face mask (94.3%), eye goggles (94.3%), gloves (93.3%), or boots (68.6%). Farmers with high levels of education were more likely to use PPE compared with farmers with low levels of education (p < 0.05). Around 92.4% of farmers wore their regular clothes during pesticide application, and 7.1% of farmers wore torn and dirty long-sleeved shirts and trousers that did not cover most parts of the body. At the time of our field visit, none of the farmers were using PPE (gloves, glasses, masks, or goggles) *(Figure 4).*

**Table 6:** Use of Protective Clothing during Pesticide Application

**Variables**	**Poor Knowledge**	**Good knowledge**	**Total (n=210)**
Gloves			
Yes	8 (4%)	6 (60%)	14 (6.7%)
No	192 (96%)	4 (40%)	196 (93.3%)
Boots			
Yes	57 (28.5%)	9 (90%)	66 (31.4%)
No	143 (71.5%)	1 (10%)	144 (68.6%)
Face and nose mask			
Yes	5 (2.5%)	7 (70%)	12 (5.7%)
No	195 (97.5)	3 (30%)	198 (94.3%)
Long-sleeved shirt and trousers			
Yes	8 (4%)	7 (70%)	15 (7.1%)
No	192 (96%)	3 (30%)	195 (92.3%)
Eyeglasses/goggles			
Yes	5 (2.5%)	7 (70%)	12 (5.7%)
No	195 (97.5)	3 (30%)	198 (94.3%)
Wearing normal clothes			
Yes	192 (96%)	2 (20%)	194 (92.4%)
No	8 (4%)	8 (80%)	16 (7.6%)
Reasons for not using PPE (multiple answers possible)			
Lack of availability	96 (45.7%)		
Too expensive	83 (39.5%)		
Uncomfortable in the local hot and humid climate	135 (64.3%)		
Slows work	92 (43.8%)		

### Pesticide storage and disposal

**Figure 5:**
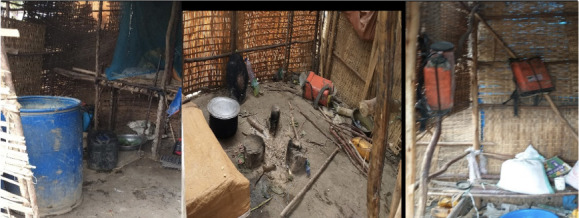
Leftover pesticides, mixer tank, and knapsacks (sprayer) in the kitchen (small huts for living and cooking)

Table 7 shows farmers' practices with regards to the storage and disposal of pesticides. A small proportion of farmers (5.7%) reported that they did not store pesticides. Around 80% of farmers with good knowledge reported directly using pesticides after purchase (pesticides were not stored). A significant number of the respondents (29.5%) kept pesticides under the bed, on the roof (23.3%), in the kitchen (8.6%) *(Figure 5)*, and near the toilet (9.5%). Only 6.2% of farmers stored pesticides in open sheds as suggested by the Ministry of Agriculture. Farmers with good pesticide knowledge were significantly less likely to store pesticides in their homes (X^2^ = 109.6, p < 0.001).

**Table 7:** Pesticide Storage and Disposal Practices (n = 210)

**Variable**	**Poor Knowledge**	**Good knowledge**	**Total (n=210)**	***X*^2^ & *P*-value**
Where do you store pesticides?				
Open shed just for pesticides	9 (4.5%)	1 (10%)	10 (4.8%)	
Toilet	20 (10%)	0	20 (9.5%)	109.66
Open field	13 (6.5%)	0	13 (6.2%)	<0.001
Roof	49 (24.5%)	0	49 (23.3%)	
Under the bed	61 (30.5%)	1 (10%)	62 (29.5%)	
Kitchen	18 (9%)	0	18 (8.6%)	
Animal shelter	26 (13%)	0	26 (12.4%)	
Do not store pesticides	4 (2%)	8 (80%)	12 (5.7%)	
What do you do with leftover pesticides?				
Dispose of in the field	14 (7%)	0	14 (6.7%)	
Mix only needed pesticides	6 (3%)	6 (60)	12 (5.7%)	67.76
Apply on other crops	179 (89.5)	3 (30%)	182 (86.7%)	<0.001
Dispose of in sewer	1 (0.5%)	1 (10%)	2 (1%)	
What do you do with empty pesticide containers?				
Dump them by the field (throw away on the farm)	183 (91.5%)	10 (100%)	193 (91.9%)	
Throw into irrigation canals or rivers (lake)	6 (3%)	0	6 (2.9%)	
Bury on farm	4 (2%)	0	4 (1.9%)	0.925
Keep for domestic uses	4 (2%)	0	4 (1.9%)	0.921
Collect and sell	3 (1.5%)	0	3 (1.4%)	
Do you rinse or clean empty containers before disposal?				
Yes	6 (3%)	0	6 (2.9%)	0.309
No	194 (97)	10 (10%)	204 (97.1%)	0.578

The majority of farmers sprayed the leftover solutions on another crop listed on the product label (86.7%) and 6. 7% of small-scale farmers disposed of them in the field. Few farmers (5.7%) reported that they mixed only the amount of pesticides they needed (bought only the needed amount of pesticides). A very few farmers stated that they discarded the leftover pesticides close to irrigation canals and drains (1%). A significant association (X^2^ = 67.69, p < 0.001) was observed between knowledge of pesticides and appropriate handling of leftover pesticides by small-scale farmers *(Table 7).* The vast majority (92%) of the small-scale farmers reported that they left the empty containers on the farm and 3% of farmers threw them near or into irrigation canals and the lake. No significant association was observed between dumping empty containers and knowledge of pesticides (X^2^ = 0.92, P > 0.05) *(Table 7).* Only 1.9% of farmers stated that they collected empty containers and bury them on the farm and about 1.4% of farmers collected empty containers for selling, and 1.9% of respondents kept the empty containers for domestic uses.

### Pesticides and biodiversity in the study area

**Figure 6:**
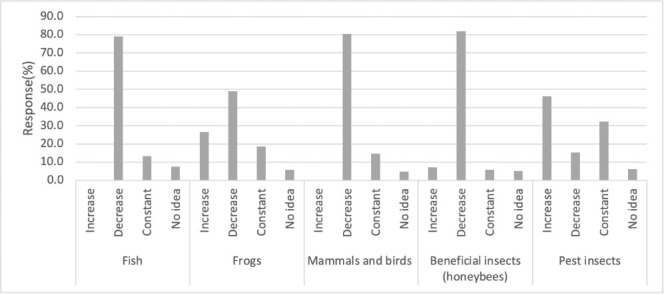
Small-scale farmers report of changes in biodiversity along the littoral zone of Lake Ziway over the last two years

About 57% of farmers reported mixing pesticides on the farm where they may be carried away into a nearby water body (e.g., Lake Ziway) by flood and 31% of farmers mixed and sprayed pesticides near water bodies. Likewise, they used water from the lake to blend pesticides in the field and wash tanks after spraying. The majority of respondents noted changes in the aquatic life around Lake Ziway following pesticide application. However, the majority of the small-scale farmers depended on their personal experience instead of following instructions on the concentration rate on pesticide labels. Overuse of pesticides in the study area poses a threat to these organisms. The survey study further showed that useful insects, fish, birds, and other animals may be decreasing in the region. When farmers were asked whether they had observed any changes in the number of fish, frogs, insects, and birds and animals in the area over the last two years after pesticide application, 79%, 49%, 81.9%, and 29.3% of the farmers reported that they had noticed a decrease in the numbers of fish, frogs, insects, and birds and animals, respectively (Figure 6). Likewise, the farmers stated that their farms are rarely visited by honeybees and a shortage of honeycombs, which were previously abundant in the study area.

## Discussion

In the present study, males performed the majority of farming activities (98%). Similarly, Waichman *et al.*^18^ and Adjrah *et al.*^6^ reported that farming was performed by males in Brazil (97.4%) and Togo (92%), respectively. An investigation by Nguetti *et al*.^19^ also showed that 90% of farmers in Kenya were male. This numerical importance of males may be due to the hardness of the work. Many of the small-scale farmers in the present study were illiterate with no formal education, and most of the small-scale farmers did not receive training on the proper use of pesticides. Many of the farmers had received informal training from untrained farmers. A study by Negatu *et al.*^9^ in the Rift Valley region in Ethiopia reported that local government extension workers and farmer cooperatives did not provide needed training to small-scale farmers on the proper usage of pesticides. In addition, there were no programs provided by local health extension services or other institutions such as the local labor and social affair or environmental office. Accordingly, these farmers could not read or understand pesticide labels about the proper and safe use of pesticides.^18^ Consequently, the application procedures were disorganized and haphazard. This has significant effects on the environment and the health of farmers. Farmers with high levels of education were well-informed about pesticide safety, and could read, recognize, and obey hazard signs on container labels, and understanding the effects of poor pesticide usage practices.^20^

Pesticide usage information in the study area is highly influenced by agrochemical retailers who are motivated by pesticide sales. The influence of sellers on small-scale farmers' pesticide applications has been previously reported in Ethiopia around the Rift Valley region^9^ and other low-income countries.^3^,^8^ This result was also in agreement with a study by Mohanty *et al.*^17^ Other sources of information on pesticides included colleagues or small informal shops, similar to a study by Nalwanga and Ssempebwa in Uganda.^21^ A great number of small-scale farmers purchased pesticides from agrochemical retailers and few retailers had a formal education about pesticides.^22^ As a result, pesticide retailers are unable to educate farmers on the appropriate use, storage, and disposal of pesticides, resulting in increased occupational and environmental hazards.

Our results demonstrate that class II pesticides such as metalaxyl, cypermethrin, lambda-cyhalothrin, profenofos, and mancozeb are the most commonly used pesticides for small-scale farmers in the study area. Fortunately, none of the pesticides reported in this study are in WHO Class I. In contrast, according to previous studies conducted in Ethiopia by Mengstie *et al.,*^2^ pesticides such as aldicarb Class 1a (extremely hazardous), imported for the flower industry, have been found on vegetable farms. Other studies have reported extended use of Class I pesticides in developing countries. For instance, in survey studies by Matthewsa *et al.*^25^ in Cameroon and Jørs *et al*.^26^ of Bolivia small-scale farmers reported that one of the regularly used organophosphorus insecticides was methamidophos, which is categorized as a highly hazardous Class 1b pesticide.^25^ The use of Class I pesticides and other banned pesticides has also been reported in Vietnam.^26^ Conversely, a survey study conducted by Ngowi *et al*.^5^,^27^ Similarly, a study by Anna *et al*.^28^ in Uganda reported that small-scale farmers primarily used Class II and III pesticides. Class II pesticides are classified as moderately hazardous and threaten human health and the environment, and thus other less dangerous options should be encouraged.^8^ In the present study, key informants such as chemical retailers and agricultural experts were interviewed in Ziway and Meki, small towns closer to the study area near Lake Ziway where pesticide retailers are found, and they reported that pesticides used in the study area were predominantly Class II (moderate toxicity) and no Class I pesticides were reported. Moreover, during our visit and observations at retailer shops, no Class I pesticides were identified.

In the present study, small-scale farmers did not report the use of banned or restricted use pesticides such as DDT or endosulfan. However, in previous studies by Negatu *et al.*^9^ in Ethiopia around the Rift Valley region, DDT and endosulfan were reported to be used by 25 and 94% of small-scale irrigation farmers, respectively, within the 12 months before the interview and by 87 and 98% of small-scale irrigation farmers, respectively, since their involvement in pesticide application work. Similarly, Mengstie *et al.*^3^ reported the use of banned pesticides such as DDT by vegetable farmers in Ethiopia around Meki, a small town located northwest of Lake Ziway, and Ziway, a town located southwest of Lake Ziway. A possible reason for the absence of these banned and restricted pesticides such as endosulfan and DDT in the present study could be attributed to the effectiveness of the National Implementation Plan (NIP) for the Stockholm Convention in Ethiopia.^29^ According to Gubena,^30^ the main aim of the NIP is to prepare comprehensive and practical activities for the effective control of persistent organic pollutant (POP) chemicals in the Ethiopian situation and to decrease, and eventually end, the use and release of POPs after fulfilling the requirements of the Stockholm Convention and national sustainable development objectives and strategies such as the Environmental Policy and Plan for Accelerated and Sustainable Development.^29^

About 45% of the farmers sprayed chemical pesticides up to 12 to 15 times. Roughly 12% of the farmers reported applying pesticides more than 15 times per season. Consequently, the rate of pesticide use by small-scale vegetable farmers was very high. This intensive use of pesticides may result in frequent contact with pesticides, which can lead to substantial human health problems and possible environmental contamination. Sun *et al.*^31^ revealed that inadequate governmental agricultural extension services are the most important factor in the overapplication and misuse of chemical pesticides. Some countries in Africa report substantial use of pesticides, for instance, in Ghana vegetable farmers sprayed more than 12 times per crop cultivation and in Tanzania, farmers sprayed more than 21 times per crop season.^27^

The present study assessed the practice of storage and disposal of pesticides by farmers and their association with the level of knowledge. The level of education had an association with the level of knowledge. This was apparent from the present study in addition to a study in South India by Mohanty *et al.*^17^ where a significant association was reported between a good level of knowledge about pesticide disposal and education level. In the present study, farmers with good knowledge had better pesticide practices than farmers with poor knowledge. Pesticide storage practices were largely inadequate, with 94.5% of farmers storing pesticides in residential rooms under the bed, on the roof, in the kitchen, in the toilet, and in animal shelters with other items *(Figure 5).* Only 5.7% did not store pesticides, as they reported that they purchased the required amount and used it immediately *(Table 7 ).* Similarly, a study conducted in the West Bank by Sa'ed *et al.*^32^ showed that few farmers (7.3%) bought and used pesticides directly (did not store). Our study found that farmers with good knowledge had better storage practices compared to farmers with poor knowledge. Our results were consistent with a study conducted in Puducherry, South India by Mohanty *et al.*^17^

Proper pesticide waste disposal is also an important part of appropriate pesticide use. Uncontrolled discharge of pesticides into the environment can threaten human health and pollute the environment.^33^ Most farmers disposed of empty containers on the farm *(Table 7).* Empty pesticide containers may retain significant amounts of pesticide solution or powder if not rinsed well. Although only 8.1% of farmers in the present study reported reusing empty containers for domestic purposes, the practice is widespread in many low-income countries. Studies by Benjamin *et al.*^8^ in Rwanda, Nadja *et al.*^34^ in Tanzania, and Jallow *et al.*^35^ in Kuwait reported that farmers used empty pesticide containers for domestic use, such as storing drinking water and food ingredients.

In the present study, nearly half of farmers did not safely dispose of leftover pesticides and disposed of leftover pesticide mixtures in the field or applied to other crops. Very few farmers reported mixing only the needed amount of pesticides. Sa'ed *et al.*^32^ reported that 29.4% of farmers stored leftover pesticides in an unlabeled plastic container and many poor farmers reused the containers as drinking vessels. The practice of discarding excess pesticide solution onto farmland in the present study was correlated with knowledge, as the majority of the farmers did not know that pesticides can affect soil and gradually leach into the surrounding water body (e.g. Lake Ziway). In the present study, the majority of small-scale farmers (97%) did not rinse or clean empty containers before disposal. Disposing of leftover pesticides and empty pesticide containers as seen in the present study is a concern because it has an impact on the environment by polluting soil, surface, and groundwater besides affecting the health of non-target creatures. These poor practices are the major concerns associated with pesticide use, management, and control in low-income countries.^36^

In the present study, very few small-scale farmers showered or bathed after pesticide mixing and spraying. Mekonnen and Agonafir^37^ and Negatu *et al.*^9^ conveyed comparable results in which many of the farmers did not habitually take a shower after the application of pesticides. Only 35% of the small-scale farmers considered wind direction during spraying. This is in agreement with the study done in Nepal.^38^ According to Khanal and Singh^39^ not considering the wind direction can result in bad odor, difficulty reaching the targeted crop with the spray, as well as inhalation by the person spraying the pesticides. Inadequate knowledge of pesticide use and method of application reported in the present study is in agreement with other studies carried out in low-income countries.^19^,^38^,^40^,^41^

None of the small-scale farmers in the present study reported using biological pesticides or other IPM methods. Integrated pest management has been shown to reduce the use of pesticides and improper practices. Integrated pest management focuses on the significance of the production of healthy crops and natural pest control systems.^27^,^42^ Even though most small-scale vegetable farmers had some knowledge of the health effects of pesticide use, they did not protect themselves effectively from acute pesticide toxicity. The farmers in the present study had poor use of PPE, leading to inadequate protection when mixing and spraying pesticides. From field observations, it was noted that none of the farmers used any PPE *(Figures 2 and 4).* Likewise, a study by Negatu *et al.*^9^ in the Rift Valley region in Ethiopia reported that less than 5% of farmers used masks or gloves during pesticide application. Three-fourths of small-scale vegetable farmers in this study reported that they did not wear boots and 94% did not wear eye protection, while 94.2% of farmers wore normal clothes while they were spraying and mixing pesticides. A study by Mekonnen and Agonafir^37^ found that 18% of small-scale farmers had ill-fitting goggles and the other 29% wore worn-out gloves. In the present study, nearly 97.5% of farmers with poor pesticide knowledge did not use face masks or gloves while spraying. This is consistent with the results of a study conducted by Williamson *et al*.^43^ Farmers reported that they did not use PPE because of inadequate knowledge about safety measures, inaccessibility of protective devices at the local market, because PPE was uncomfortable in the local hot and humid climate, and high cost at private shops. Warm climate was one of the causes of low use of PPE in a study in the United States.^44^

Small-scale farmers tended to use large amounts of pesticides to protect their vegetables from pests to increase crop production.^6^ Our results indicate that the rate of pesticide application is higher. In the present study, no farmers reported correct knowledge regarding pesticide application interval. Similarly, extensive pesticide application has been reported in other countries: in Benin, many farmers spray pesticides every 3–5 days^43^ and in Brazil, the pesticide spraying rate ranged from 3–15 days.^18^ Application of insecticides and fungicides dominated chemical pest management in the vegetable farms in the study area. However, very few farmers reported the use of herbicides because most of the time vegetable farmers in the study area used inexpensive human labor to remove weeds manually. In North America and Europe, herbicides are the most commonly used pesticides followed by insecticides, fungicides, and others. This is probably because it is less expensive than renting additional labor for weeding.^18^

The most common health problems related to exposure to agrochemicals reported by small-scale farmers include skin problems, headache, teary and eye irritation, seizure, sore throat, and respiratory disorder, fatigue, nausea, and stomachache. Burning sensation in the eyes was the most regularly reported symptom. According to the United States Environmental Protection Agency (USEPA),^45^ all of the above symptoms could be indications of pesticide exposure, as most of these symptoms are considered to be common manifestations of acetylcholinesterase inhibition.^46^ Many of the reported pesticides in the present study have acute toxicity to humans. For example, cypermethrin is moderately toxic through skin contact or ingestion. It may also irritate the skin and eyes. Symptoms of dermal exposure include unresponsiveness, stinging, itching, burning sensation, loss of bladder control, uncoordination, seizures, and possible death.^46^ According to Khan *et al.*,^47^ exposure to lambda-cyhalothrin caused up to 20% and 57.1% of inhibition in the activity of the brain cholinesterase enzyme. Lake Ziway is a key source of water supply for irrigation and domestic purposes such as drinking, food preparation, and cleaning clothes in addition to its crucial role in the surrounding ecosystem. Misuse and improper handling of pesticides presents possible health risks to the communities around Lake Ziway as well as the biodiversity of the lake, which may be exposed to water polluted by pesticides carried by flooding. Moreover, in addition to work-related exposures,^48^ human exposure to pesticides can occur through exposure to polluted water sources.^49^

Pesticides have been found to contaminate Lake Ziway.^13^,^50^,^51^ These pesticide residues are highly lethal to fish and amphibians.^52^ The present study revealed insufficient knowledge of small-scale farmers about the fate of pesticide accumulation in surface water and groundwater. Anju *et al.*^53^ examined the harmful effects of pesticides on aquatic organisms, including fish. In the present study, above 86% of the farmers interviewed sprayed pesticides close to Lake Ziway. Similarly, they used water from the lake to blend pesticides in the farm, as well as cleaning and rinsing the spraying container after spraying tasks. This misuse of pesticides may intensify the susceptibility of the farmers to pesticide poisoning as well as affecting water quality. Likewise, 46% of the respondents received information or advice from other farmers and personal experience instead of paying attention to the concentration rate on pesticide labels. Increasing misuse of these pesticides in the study area is a probable cause of the deterioration of aquatic organisms.

The study further revealed that useful insects, birds, and fish are declining in the study area. The farmers reported changes in the number of insects and animals in the area over the last two years following pesticide application, and 89%, 79%, and 80% of farmers stated that they had noticed a decrease in the numbers of beneficial insects, frogs, and birds and mammals, respectively *(Figure 3).* These declines may be due to exposures linked to the misuse of pesticides by farmers.^54^,^55^ Similarly, farmers reported a lack of honeybees on their farms, which used to be plentiful in the area. A possible explanation could be the use of pesticides such as organochlorine, carbamate, organophosphorus, and parathyroids.^56^

There are a few limitations to the present study. Long-term and chronic well-being disabilities were not addressed due to methodological problems. In addition, quantitative measures of neuron-electrophysiology evaluating the health effects of long-term pesticide exposure such as blood chemistry testing, electrolyte levels, vitamin levels, etc., were not investigated. Furthermore, the time and extent of pesticide exposure were not sufficiently estimated in the present study.

## Conclusions

In contrast to findings in previous studies, small-scale vegetable farmers around the Lake Ziway watershed in Ethiopia did not use the most hazardous WHO class 1a and 1b pesticides and banded organochlorine pesticides including DDT and endosulfan. The WHO class II pesticides were the most commonly used agrochemicals in the study area. However, the present study revealed that farmers lacked sufficient knowledge of appropriate pesticide usage and storage. Lack of knowledge included limited use of PPE and inappropriate storage and disposal of pesticides. This may lead to acute intoxication, chronic health problems, and environmental degradation. There is a need to increase farmers' knowledge and awareness of pesticides by providing training on the health-related effects of pesticide exposures, the impact of pesticides on the environment, as well as the proper disposal and storage of pesticides. Efforts are needed to enhance the decision-making capability of local people by promoting IPM methods as alternatives to chemical pesticides. To mitigate the health effects of the misuse of pesticides, there is a need for continuous pesticide safety education for farmers on the use of PPE, personal hygiene, and sanitation practices during and after spraying. Training pesticide retailers to improve their awareness of pesticides is also important as they are farmers' key sources of information concerning pesticides.
