# Supplementary material for: Small-scale Farmer Pesticide Knowledge and Practice and Impacts on the Environment and Human Health in Ethiopia
Source: J Health Pollut. 2021 May 28;11(30):210607. doi: 10.5696/2156-9614-11.30.210607 (PMC8276729; doi:10.5696/2156-9614-11.30.210607)
Supplement: Supplementary file 1 [file Mergia_Supplemental_Material_1.docx]

**Supplemental Material 1**

**Questionnaire**

**Structured and semi-structured interview questionnaire schedule for small-scale farmers**

Objective: To study how pesticides are used by small-scale farmers and the impact they have on people living close to Lake Ziway and the environment in which they are used. You have been requested to participate in this survey because I believe that you can give me ideas, information, and views on issues related to knowledge of pesticides, practices, and their effect on your health and the surrounding environment. Your kind cooperation in providing an interview is highly appreciated. I want to assure you that the information you give me will be completely confidential and will be used exclusively for our study, and I will not be taking down your name so your answers will be anonymous.

Note: General information

Date__/___/______ Name of village …………………………………………………………………....................................................

Name of the district:

1. Adami-Tulu-Jido-Kombolcha (Ziway area) B. Dugda (Meki area)

Additional observations during the interview -…………………………...................................................……………………………………………………………………………………………………………………………………………………………...................................................…………………………………………………………………...................................................…………………………………………………………………...................................................…………………………………………………………………...................................................…………………………………………………………………...................................................…………………………………………………………………...................................................………………………………………………………………….......

**Section I: Demographic characteristics of small-scale farmers**

1. Sex A. Male B. Female
2. Age: A 18 – 30 B. 31-54 C. 55 and above
3. Socio-economic background of small-scale vegetable farmers

| \| Items \|  \|  \| \| --- \| --- \| --- \| | Response | Remarks |
| --- | --- | --- | --- | --- | --- |
| Education level   1. None (Illiterate) 2. 1 to 8 grades 3. 9 to 12 grades 4. College/University |  |  |
| Farm sizes   1. Less than one hectare 2. Greater than one hectare |  |  |
| Land tenure situation   1. Landowners 2. Landholders (rented from owners for 3- 5-year contracts) |  |  |
| Trend pesticide use past 5 years   1. Increased 2. Constant |  |  |
| Farming experience   1. 1-3 years 2. 5-10 years 3. > 10 years |  |  |
| Professional training   1. Yes 2. No |  |  |

1. What type(s) of pesticide(s) do you apply? ……………………………………………………………………………………………………………………………………………………………………………………………………………………………………………………………………………………………………………………………………………………………………………………………………………………………………………………

**Section II: Farmers' knowledge, attitude, and understanding of pesticides**

1. Do you think that pesticides affect human health?
2. Yes
3. No
4. Don't know
5. Do you think that pesticides affect the environment?
6. Yes
7. No C. Don't know
8. Do you think pesticides are indispensable for high crop yield?
9. Yes
10. No
11. Don't know
12. Do you read, understand and follow pesticide labels?
13. Yes
14. No
15. How do pesticides enter the human body? (Multiple answers possible)
16. Dermal B. Inhalation C. Oral D. Eyes E. Don't know
17. Do you know pesticides that are banned or restricted for use?
18. Yes
19. No
20. Do you know the reasons for banning or restricting pesticides?
21. Highly toxic B. Not effective C. Expensive D. Don't know
22. Are you aware of the toxicity color codes present on the pesticide containers?
23. Yes B. No

**Section III: Sources of pesticides and pesticide information**

1. Where do you get pesticide information for purchase or usage?
2. Agrochemical retailers B. Other farmers C. Previous experience D. Agriculture extension officers
3. Source of pesticide dose information (how do you determine the dose of the pesticides you apply?)
4. Agrochemical retailers B. Other farmers C. Previous experience/ information on container D. Agriculture extension officers
5. Place where pesticides were purchased
6. Agrochemical retailers B. General household shops C. Other farmers

**Section IV: Pesticide use practices**

1. Do you apply any pesticides on your farm?

A. Yes B. No

1. Do you use biological pesticides or other forms of integrated pest management?

A. Yes B. No

1. Do you drink while mixing or spraying?

A. Always B. Sometimes C. Never

1. Do you eat while mixing or spraying?

A. Always B. Sometimes C. Never

1. During the preparation of the different formulae, do you use standard pesticide quantification materials?
2. Yes B. No
3. Do you spray following the wind direction?

A. Always B. Sometimes C. Never

1. How often do you apply this/these pesticide(s)? (Frequency of pesticide application)
2. 3-5 times per season B. 7-10 times per season C. 12-15 times per season D. More than 15 times per season
3. How much pesticide do you apply per ha? …………………………………………………………………………………………………………………………………………………
4. Do you take shower immediately after mixing or spraying?
5. Always B. Sometimes C. Never
6. Where are pesticides mixed?
7. On the farm
8. Near water/community water sources
9. At home
10. What equipment do you use to mix pesticides?
11. Knapsacks B. Blue containers (drum)
12. How do you mix pesticides during formulation?
13. With a stick, but bare hands
14. With bare hands
15. With hands and wearing gloves

**Section V: Symptoms frequently experienced after pesticide spraying procedures**

1. Have you ever felt any discomfort/illness after pesticide application?
2. Yes B. No C. Sometimes D. Don't know
3. If yes, what were your symptoms?
4. Let respondent give an answer and then mark down alternative answers: do not prompt with possibilities. *(multiple answers possible)*

| Symptoms | Responses | Marks |
| --- | --- | --- |
| Dizziness/headaches |  |  |
| Itchy skin |  |  |
| Skin burning sensation |  |  |
| Runny nose |  |  |
| Coughing |  |  |
| Teary eyes/eye irritation |  |  |
| Excessive sweating |  |  |
| Dry/sore throat |  |  |
| Shortness of breath/cough |  |  |
| Stomach pain, cramps, and diarrhea |  |  |
| Skin redness/white patches |  |  |
| Poor vision |  |  |
| No health impairment |  |  |
| Fatigue |  |  |
| Nausea |  |  |
| Vomiting |  |  |

**Section VI: Use of personal protective equipment (PPE)**

1. Do you wear protective clothing when applying/mixing pesticides?

| Variable | Response | Comment |
| --- | --- | --- |
| Glove |  |  |
| Yes |  |  |
| No |  |  |
| Boots |  |  |
| Yes |  |  |
| No |  |  |
| Face and nose mask |  |  |
| Yes |  |  |
| No |  |  |
| Long-sleeved shirt and trousers |  |  |
| Yes |  |  |
| No |  |  |
| Eyeglasses/goggles |  |  |
| Yes |  |  |
| No |  |  |
| Wear normal clothes |  |  |
| Yes |  |  |
| No |  |  |
| Reasons for not using PPE (multiple answers possible) |  |  |
| Lack of availability |  |  |
| Too expensive |  |  |
| Uncomfortable in the local hot and humid climate |  |  |
| Slows work |  |  |

**Section VII: Pesticide storage and disposal**

1. Where do you store pesticides?
2. Open shed just for pesticides B. Open field C. Under the bed D. Kitchen
3. Toilet F. Roof G. Animal shelter H. Do not store pesticides
4. What do you do with unused leftover pesticides?
5. Dispose of in the field B. Apply on other crops
6. Mix only needed pesticides D. Dispose of in sewer
7. What do you do with empty pesticide containers?
8. Dump them by the field (throw away on the farm) B. Throw into irrigation canals or rivers
9. Bury on-farm D. Keep for domestic uses E. Collect and sell
10. Do you rinse or clean empty containers before disposal?

A. Yes B. No

**Section VII. Pesticide use and biodiversity in the study area**

1. Have you observed any changes in the number of fish, frogs, beneficial insects, insect pests, birds, and animals in the area over the last two years after pesticide application?

| Biota | Response |  |
| --- | --- | --- |
| Fish |  |  |
| Increased |  |  |
| Decreased |  |  |
| Constant |  |  |
| Frogs |  |  |
| Increased |  |  |
| Decreased |  |  |
| Constant |  |  |
| Birds |  |  |
| Increased |  |  |
| Decreased |  |  |
| Constant |  |  |
| Mammals |  |  |
| Increased |  |  |
| Decreased |  |  |
| Constant |  |  |
| Beneficial insects (honeybee) |  |  |
| Increased |  |  |
| Decreased |  |  |
| Constant |  |  |
| Insect pests |  |  |
| Increased |  |  |
| Decreased |  |  |
| Constant |  |  |
