# Supplementary material for: Small-scale Farmer Pesticide Knowledge and Practice and Impacts on the Environment and Human Health in Ethiopia
Source: J Health Pollut. 2021 May 28;11(30):210607. doi: 10.5696/2156-9614-11.30.210607 (PMC8276729; doi:10.5696/2156-9614-11.30.210607)
Supplement: Supplementary file 2 [file Mergia_Supplemental_Material_2.docx]

**Supplemental Material 2**

Table 1: Types of Pesticides (Fungicides) Used by Small-Scale Vegetable Farmers near Lake Ziway, Ethiopia

| Trade name | Active ingredient(s) and concentration | Chemical  Class | Reported use (N) | Reported use (%) | WHO toxicity class^56^ |
| --- | --- | --- | --- | --- | --- |
| Fungicides |  |  |  |  |  |
| Agrolaxyl M2-63.5WP | Metalaxyl – Mancozeb | Dithiocarbamate | 206 | 98.1 | Unk |
| Bayleton 25WP | Triadimegon 250 g/kg |  | 82 | 39.0 | II |
| Cerozeb 80WP | Mancozeb 800 g/kg | Dithiocarbamate | 210 | 100.0 | II |
| Cropaxyl Gold 72WP | Metalaxyl 80 g/kg + Mancozeb 640 g/kg | Phenyl amide + Dithiocarbamate | 206 | 98.1 | Unk |
| Cropzeb 80WP | Mancozeb wp 800 g/kg | Dithiocarbamate | 210 | 100.0 | II |
| Cruzate R | Cymoxanil + copper oxychloride |  | 96 | 45.7 | Unk |
| Ethiozeb | Mancozeb 80% wp | Dithiocarbamate | 210 | 100.0 | U |
| Fungozeb | Mancozeb 80% wp | Dithiocarbamate | 96 | 45.7 | U |
| Indofil M-45 | Mancozeb 80% wp | Dithiocarbamate | 210 | 100.0 | U |
| Indom | Mancozeb 80% wp | Dithiocarbamate | 196 | 93.3 | U |
| Kocide 101 | Copper hydroxide | Oxime+inorganic | 79 | 37.6 | II |
| Mancolaxyl 72WP | Mancozeb 80% + metalaxyl 64% | Dithiocarbamate+ | 201 | 95.7 | Unk |
| Mancolaxyl 72%WP | Metalaxyl 85 + mancozeb 64% | Dithiocarbamate | 210 | 100.0 | II |
| Masco 8-64 | Mancozeb 64% WP | Dithiocarbamate | 96 | 45.7 | II |
| Matco 8-64 | Metalaxyl 8% + mancozeb 64% WP | Dithiocarbamate | 205 | 97.6 | II |
| Natura 250EW | Tebuconazole |  | 56 | 26.7 | II |
| Nimrod 25EC | Buprimate |  | 72 | 34.3 | II |
| Perfecto175SC | Imidacloprid 12.5% + lambada cyhalothrin 5% | Neonicotinoid+ Pyrethroid | 69 | 32.9 | Unk |
| Revus 250SC | Mandipropamid | Triazole Fungicide | 76 | 36.2 | II |
| Ridomil 68WG | Metalaxyl-M 68% WG | Phenyl amide | 207 | 98.6 | II |
| Sabozeb 80%WP | Mancozeb 800 g/kg | Dithiocarbamate | 201 | 95.7 | II |
| Tilt@250 | Propiconazole | Triazolefungicides | 96 | 45.7 | II |
| Unizeb | Mancozeb 80% wp | Dithiocarbamate | 206 | 98.1 | II |
| Victory 72 WP | Metalaxyl + mancozeb 64% | Phenyl amide + Dithiocarbamate | 203 | 96.7 | Unk |

Table 1S continued: Types of Pesticides (Insecticides) Used by Small-scale Vegetable Farmers near Lake Ziway, Ethiopia

| Trade Name | Active ingredient(s) and concentration | Chemical  Class | Reported use (N) | Reported use (%) | WHO toxicity class ^56^ |
| --- | --- | --- | --- | --- | --- |
| Agro-Thoate40%EC | Dimethoate 40% EC | Organophosphate | 203 | 96.7 | II |
| Aim 10%EC | Alpha-cypermethrin 100 g/l | Pyrethroid | 196 | 93.3 | II |
| Con-Findence | Imidacloprid 35% W/V | Neonicotinoid | 109 | 51.9 | II |
| Coragen 200SC | Chlorantraniliprole | Anthranilic diamide | 96 | 45.7 | III |
| Decis 2.5%EC | Deltamethrin | Pyrethroid | 153 | 72.9 | II |
| Dimeto 40%EC | Dimethoate | Organophosphate | 156 | 74.3 | II |
| Dursban 48%EC | Chlorpyrifos-ethyl | Organophosphate | 96 | 45.7 | II |
| Ethiodemethrin2.5EC | Deltamethrin 25 g/l | Pyrethroid | 125 | 59.5 | II |
| Ethiolathion 50EC | Malathion | Organophosphate | 93 | 44.3 | II |
| Ethiothoate 40%EC | Dimethoate | Organophosphate | 165 | 78.6 | II |
| Ethiozinon 60EC | Diazinon | Organophosphate | 97 | 46.2 | II |
| Farrate | Lambda-cyhalothrin 5% | Pyrethroid | 203 | 96.7 | II |
| Globe 72%EC | Profenofos 720 ml/l | Organophosphate | 201 | 95.7 | II |
| Hanclopa 48%EC | Chlorpyrifos | Organophosphate | 56 | 26.7 | II |
| Helerat 50EC | Lambda-cyhalothrin | Pyrethroid | 197 | 93.8 | II |
| Karate 2.5%EC | Lambda-cyhalothrin 25 ml/L | Pyrethroid | 197 | 93.8 | II |
| Karate 5%EC | Lambda-cyhalothrin 50 ml/l | Pyrethroid | 197 | 93.8 | II |
| Lamdex 5EC | Lambda-cyhalothrin | Pyrethroid | 96 | 45.7 | II |
| Perfecto@175SC | Imidacloprid 12.5% + Lambda-cyhalothrine 5% | Neonicotinoid+pyrethroid | 186 | 88.6 | Unk |
| Polytrin_KA315EC | Profenofos 300 g/l + Lambda-cyhalothrine 15 g/l | Organophosphate | 138 | 65.7 | Unk |
| Profit 72EC | Profenofos 72% EC | Organophosphate | 205 | 97.6 | II |
| Pyrinex 48%EC | Chlorpyrifos-ethyl | Organophosphate | 82 | 39.0 | II |
| Radiant 120SC | Spinetoram | Spinosyn | 54 | 25.7 | II |
| Roger | Dimethoate 40% EC | Organophosphate | 167 | 79.5 | II |
| Selecron 720EC | Profenofos ‘‘Q’’ 720 g/l | Organophosphate | 203 | 96.7 | II |
| Tracer 480SC | Spinosad | Spinosyn | 67 | 31.9 | II |
| Tutan 36%SC | Chlorfenapyr 360 g/l | Organophosphate | 68 | 32.4 | II |
| Hanclopa 48%EC | Chlorpyrifos | Organophosphate | 53 | 25.2 | II |
| Herbicides |  |  |  |  |  |
| Herbax | Propanil 360 g/L | Acetanilide | 3 | 1.4 | III |
| Aura 72SL | 2,4-D 720 g/l | Chlorophenoxy | 5 | 2.4 | II |

*Toxicity class as classified by the World Health Organization,^56^ where Ia, extremely hazardous; Ib, highly hazardous; II, moderately hazardous; III, slightly hazardous; U, unlikely to present acute hazard in normal use; unk, unknown*
